# Explainable AI-Based Analysis of Deflection in RC Beams with Longitudinal GFRP Bars in Tension Zone

**DOI:** 10.3390/polym18060728

**Published:** 2026-03-17

**Authors:** Muhammet Karabulut

**Affiliations:** Department of Civil Engineering, Zonguldak Bulent Ecevit University, Zonguldak 67100, Türkiye; karabulut@beun.edu.tr

**Keywords:** GFRP-reinforced concrete beams, deflection behavior, cracking behavior, SHAP analysis, explainable artificial intelligence, sensitivity analysis, creep effects

## Abstract

The research gap addressed in this study is the lack of a transparent and quantitative evaluation of the governing parameters influencing deflection behavior in reinforced concrete (RC) beams reinforced with glass fiber-reinforced polymer (GFRP) bars. The objective of this study is to identify and quantify the relative importance of the key parameters controlling deflection in GFRP-reinforced RC beams, which exhibit fundamentally different behavior compared to steel-reinforced beams due to the linear-elastic response of GFRP bars until rupture. To achieve this objective, the method integrates explainable artificial intelligence (XAI) techniques, including SHapley Additive exPlanations (SHAP), Pearson correlation heatmap, scatter plot analysis, and sensitivity analysis—with experimental structural data obtained from beams with three different concrete strength classes. The main contribution of this study is the quantitative ranking and interpretation of the governing parameters affecting deflection behavior through a transparent and data-driven framework. Key parameters—including elastic modulus (Ec), compressive strength (fck), creep coefficient (φ), failure moment (Mexp), effective moment of inertia (Ieff), and applied load (P)—were evaluated. The results consistently indicate that stiffness- and capacity-related parameters dominate the deflection response. Sensitivity analysis reveals that the failure moment (Mexp) is the most influential parameter, contributing approximately 23% of the total relative influence on deflection, followed by compressive strength (fck) and cracking-related parameters. Pearson correlation heatmap and scatter plot analyses further confirm strong relationships between deflection and Ec, fck, φ, and Ieff. The proposed framework improves the interpretability of deflection prediction in GFRP-reinforced RC beams and provides a transparent basis for serviceability-based structural design and performance-oriented assessment.

## 1. Introduction

Recent developments in structural engineering research have increasingly emphasized the importance of accurately predicting the serviceability performance of FRP-reinforced concrete beams, particularly with respect to deflection behavior under short- and long-term loading conditions. In this context, machine learning-based frameworks have demonstrated strong capability in predicting the deflection response of lightweight foamed concrete T-beams and GFRP-reinforced continuous beams, offering improved accuracy compared to conventional empirical formulations [1,2,3].

The robustness and generalization performance of ML-driven deflection models have been further investigated through systematic evaluation of training–testing data variation, nonlinear load–deflection relationships, and different reinforcement configurations in both FRP- and steel-reinforced concrete beams [4,5]. Moreover, metaheuristic optimization techniques and categorical boosting algorithms have enhanced prediction accuracy while maintaining interpretability, particularly when combined with explainable artificial intelligence tools [6,7].

Optimized machine learning approaches integrated with SHAP-based interpretation have also been successfully employed for predicting the flexural strength and overall serviceability response of FRP-reinforced concrete beams, thereby bridging the gap between data-driven prediction and mechanical understanding [8,9]. In addition, ML models have been developed to estimate crack width development and bond-dependent coefficients in GFRP-reinforced beams, which are critical parameters governing stiffness degradation and deflection growth [10,11].

Beyond flexural capacity, explainable machine learning models have been extended to evaluate punching shear capacity and serviceability performance of FRP bar-reinforced concrete flat slabs and beam systems, highlighting the versatility of AI-assisted structural assessment methodologies [12,13].

Parallel to data-driven research, extensive experimental programs have examined the flexural behavior and load–deflection response of GFRP- and FRP-reinforced concrete beams. These studies consistently report lower elastic stiffness and higher serviceability deflections compared to conventional steel-reinforced systems due to the relatively lower modulus of elasticity of FRP bars [14,15,16]. Detailed experimental investigations into cracking characteristics and mid-span deflection behavior have further demonstrated the influence of reinforcement ratio, bond performance, and reinforcement stiffness on crack spacing and deflection control [17,18,19].

Comparative studies combining experimental testing with predictive modeling have validated the flexural strength, stiffness response, and serviceability performance of beams reinforced with glass FRP bars and hybrid steel–FRP composite reinforcement, providing valuable insights for performance-based design [20,21].

Time-dependent deformation behavior remains one of the most critical aspects of FRP-reinforced concrete members. Numerous studies have examined creep-induced curvature, sustained-load deflection growth, and long-term serviceability performance under different environmental conditions [22,23,24]. These investigations highlight the compounded effects of concrete creep, shrinkage, and the relatively linear-elastic behavior of FRP reinforcement on long-term stiffness degradation.

To address these challenges, simplified analytical formulations and rational long-term prediction models have been proposed to estimate time-dependent curvatures and deflections of FRP-reinforced concrete beams with improved computational efficiency and practical applicability [25,26,27]. Environmental exposure conditions, sustained loading duration, and material properties such as elastic modulus and reinforcement ratio have been identified as dominant parameters influencing long-term deflection behavior in GFRP-reinforced systems [28,29,30].

Theoretical studies on hybrid and partially interacting FRP–concrete systems have further contributed to understanding long-term deflection mechanisms, particularly in composite and strengthened members where bond-slip and partial interaction effects play a significant role [27,31,32].

Load–deflection responses of multi-span beams, FRP-plated concrete members, and externally FRP-strengthened RC beams with anchorage systems have been analytically and experimentally evaluated to capture stiffness degradation, anchorage efficiency, and full-range nonlinear response up to failure [33,34,35]. These investigations provide deeper insight into structural ductility, redistribution capacity, and design implications.

Material innovations have also expanded the research scope, including the use of recycled aggregate concrete, lightweight concrete, and early-age concrete in combination with GFRP reinforcement. Such studies have assessed their influence on flexural stiffness, bond characteristics, and short-term deflection performance [36,37,38]. The integration of alternative materials highlights the growing emphasis on sustainability alongside structural performance.

Furthermore, experimental research examining the static and dynamic behavior of RC beams strengthened with GFRP and CFRP systems under progressive damage has clarified the interaction between strengthening techniques and serviceability performance, including deflection control and stiffness retention [21,39].

Comprehensive reviews and state-of-the-art assessments have synthesized experimental findings, analytical developments, and emerging machine learning applications related to flexural behavior, cracking control, deflection prediction models, and code-based design implications for FRP-reinforced concrete beams. These reviews identify key governing parameters, highlight discrepancies among existing design provisions, and emphasize the need for unified serviceability criteria tailored to FRP-reinforced systems [40,41]. The load–deflection behavior of various polymer-based materials under three-point bending tests has been widely investigated in the literature [42,43], highlighting the flexural performance and mechanical response characteristics of different composite polymer systems.

Recent studies have investigated the flexural performance of FRP-reinforced ultra-high-performance concrete (UHPC) structural elements, showing that combining advanced fiber-reinforced composites with UHPC can significantly improve flexural capacity, crack control, and durability. Experimental investigations have also examined the fatigue behavior of FRP-reinforced UHPC members under repeated loading, providing important insights into their long-term structural performance. These findings highlight the growing interest in innovative material systems and advanced analytical approaches for enhancing structural efficiency and reliability. In this context, the use of data-driven and interpretable modeling techniques offers a promising framework for understanding complex structural responses and identifying the governing parameters influencing deflection behavior [44,45].

In this study, the effectiveness of the parameters influencing the deflection capacity of reinforced concrete beams incorporating longitudinal GFRP reinforcement in the tensile zone was investigated using SHAP analysis. A distinctive aspect of the research is the evaluation of which parameters play a more dominant role in GFRP-reinforced beams produced with low-, medium-, and high-strength concrete, based on artificial intelligence-assisted analyses and supported by three-point bending test results.

Additionally, Pearson correlation heatmaps and scatter plots were generated to examine the relationships among variables. Frequency–cumulative distribution graphs were also analyzed to provide a comprehensive assessment of the data distribution and parameter influence.

This study investigates reinforced concrete (RC) beams with GFRP longitudinal reinforcement in the tension zone, and the application of XAI to deflection prediction for this type of beam demonstrates the suitability of XAI for estimating the structural deflection capacity corresponding to applied loads. In addition, the effects of different concrete strength classes and material properties on deflection behavior in such beams have been evaluated based on experimental results. In this context, XAI represents an innovative approach, and its application to the structural behavior prediction of RC beams with different characteristics is expected to contribute to the existing literature. In addition, the relative contributions of the parameters influencing deflection were investigated, and their respective percentages were determined. Accordingly, the degree to which each parameter affects deflection behavior has been clearly identified through the proposed XAI framework.

Although there are studies addressing some of the individual subtopics, there is no study that directly covers the key aspects of this subject comprehensively.

However, the number of studies that simultaneously consider the following three aspects remains quite limited:

- Reinforced concrete beams with longitudinal GFRP bars located in the tension zone;
- Investigation of beam deflection and flexural behavior;
- Artificial intelligence and explainable AI-based analysis incorporating different concrete strength classes effect on deflection.

## 2. Materials and Methods

### 2.1. GFRP Material

**Figure 1:**
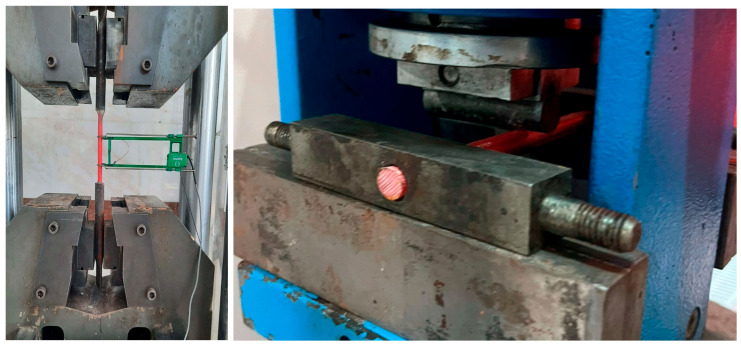
Tensile and flexural tests of GFRP specimens [3,4].

In this study, the flexural behavior of concrete beams with GFRP bars as longitudinal reinforcement in the tension zone is investigated; furthermore, the mechanical properties of the GFRP material, together with other parameters affecting deflection, and their influence on the deflection capacity are also examined. In this context, the three-point bending test results reported in the literature, specifically the data presented in reference [3], will be considered, and the artificial intelligence-assisted SHAP and other analyses conducted in this study will be evaluated accordingly. The tensile and flexural tests performed to determine the mechanical strength properties of the GFRP material used in reference [3,4,42] are presented in Figure 1, and the corresponding results are summarized in Table 1 below.

The mechanical performance of the GFRP bars employed as longitudinal reinforcement was determined through a comprehensive experimental program consisting of both standardized three-point bending tests and direct uniaxial tensile tests. The flexural response was evaluated using specimens with a total length of 150 mm and a clear support span of 100 mm, subjected to monotonically increasing load until the ultimate bending capacity was reached, beyond which a rapid reduction in load-carrying capacity was observed. Complementarily, the tensile properties were characterized through displacement-controlled uniaxial tests conducted in accordance with ASTM D7205. These tests utilized precision-engineered gripping systems designed to prevent slippage and avoid premature failure at the grip regions. Each tensile specimen was prepared with a calibrated gauge length of 400 mm and a clear distance of 140 mm between fixtures, while axial load–elongation responses were continuously monitored and recorded up to the point of rupture. Figure 1 shows the tensile and flexural tests of the GFRP specimens [3,4].

The experimentally obtained mechanical parameters are summarized in Table 1.

### 2.2. Concrete Material

Within the scope of this study, the concrete strength values affecting the deflection of GFRP-reinforced concrete beams and used in the artificial intelligence-assisted SHAP analyses were adopted from the experimental study reported in reference [3].

The 28-day compressive strength results of the low-strength (C20), medium-strength (C30), and high-strength (C40) concrete cube specimens are presented in Table 2. The cube specimens were cast in standard molds with dimensions of 150 × 150 × 150 mm.

Table 2 presents the concrete strength values of the beams considered in this study, within the scope of investigating the effectiveness of parameters influencing the deflection of GFRP-reinforced beams.

### 2.3. Three Point Bending Test

The three-point bending test is a widely used experimental method to evaluate the flexural behavior of reinforced concrete (RC) beams by applying a concentrated load at midspan while the specimen is simply supported at two end points. This configuration creates a constant bending moment region at midspan, allowing the assessment of structural response under flexural loading. The primary purpose of this test is to characterize the load–deflection behavior, flexural strength, stiffness, cracking response, and ultimate load-carrying capacity of RC beams, including those reinforced with GFRP bars.

During the test, the applied load and the corresponding midspan deflection are continuously recorded, typically using a load cell and a Linear Variable Differential Transformer (LVDT). These measurements enable the construction of the load–deflection curve, which provides essential information regarding the elastic and inelastic behavior of the beam, stiffness degradation, and failure characteristics. The flexural stiffness k of the beam can be expressed as the ratio of the applied load P to the corresponding midspan deflection δ in (1):

(1) $$k=\frac{P}{\delta }$$

One of the most critical parameters obtained from the three-point bending test is the ultimate load capacity $P_{u}$, which corresponds to the maximum load sustained by the beam prior to failure. Based on this value, the ultimate flexural moment capacity $M_{u}$ can be calculated using the following relationship in (2):

(2) $$M_{u}=\frac{P_{u}\times L}{4}$$

where L is the clear span length between the supports.

In addition, the first cracking load $P_{cr}$ can be identified from the load–deflection response as the point at which the stiffness begins to decrease due to the initiation of tensile cracks in the concrete. The corresponding cracking moment $M_{cr}$ is determined in (3) as:

(3) $$M_{cr}=\frac{P_{cr}\times L}{4}$$

**Figure 2:**
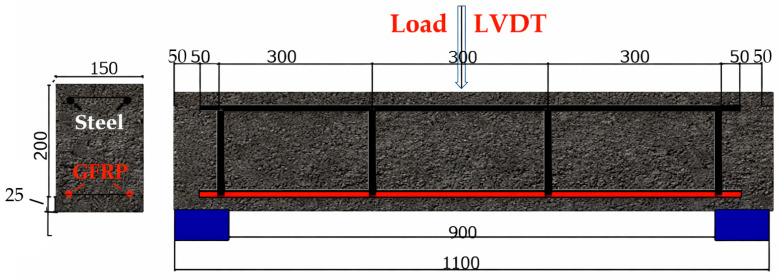
Three point bending tests of GFRP RC beams.

Figure 2 presents the geometric and reinforcement details of the GFRP RC beams, as well as the three-point bending test configuration, corresponding to the beams whose deflection values were investigated in this study.

The load–deflection relationship also provides insight into the flexural rigidity and deformation capacity of the beam. For GFRP-reinforced concrete beams, this test is particularly important because GFRP reinforcement exhibits linear elastic behavior up to failure without yielding, unlike conventional steel reinforcement. Therefore, the three-point bending test allows for the evaluation of the influence of GFRP mechanical properties, concrete strength, and reinforcement characteristics on the deflection behavior, stiffness, cracking performance, and ultimate flexural capacity of the beam.

### 2.4. SHAP (SHapley Additive exPlanations) Analysis

SHAP analysis (SHapley Additive exPlanations) is an explainable artificial intelligence (XAI) method used to interpret and quantify the contribution of each input feature to the output prediction of a machine learning model. It is based on Shapley values derived from cooperative game theory, which fairly distribute the prediction outcome among the input features according to their individual contributions.

In SHAP analysis, each feature is assigned to a SHAP value that represents the magnitude and direction of its influence on the model prediction. A positive SHAP value indicates that the feature increases the predicted outcome, while a negative SHAP value indicates that the feature decreases it. The sum of all SHAP values, together with the model’s baseline value, equals the final prediction.

Mathematically, the SHAP value $\phi_{i}$ for a feature i is defined in (4) as [46]:

(4) $$\phi_{i}=\sum_{S\subseteq F\setminus \{i\}}\frac{|S|!(|F|-|S|-1)!}{|F|!}\left[f(S\cup \{i\})-f(S)\right]$$

where

- F is the full set of features;
- S is a subset of features excluding feature i;
- f(S) is the model prediction using features in subset S;
- $\phi_{i}$ represents the contribution of feature i to prediction.

SHAP analysis provides both global and local interpretability. Global interpretability identifies the overall importance and influence of features across the entire dataset, while local interpretability explains individual predictions. This method is widely used in engineering applications to understand the influence of input parameters on structural performance, such as predicting deflection, strength, or failure behavior of reinforced concrete beams.

## 3. Results

In Reference [3], the experimental deflection values obtained from three-point bending tests of reinforced concrete beams reinforced with GFRP bars in the tension zone are considered, and the parameters affecting the deflection response are determined and interpreted in this section using artificial intelligence-assisted SHAP analysis, Pearson correlation heatmaps, and scatter plots. In this study, the ultimate deflection values corresponding to the failure load of beams with different concrete strength classes were estimated, and the influence of the governing parameters affecting these ultimate deflection values was investigated.

### 3.1. Three-Point Bending Test Results

**Figure 3:**
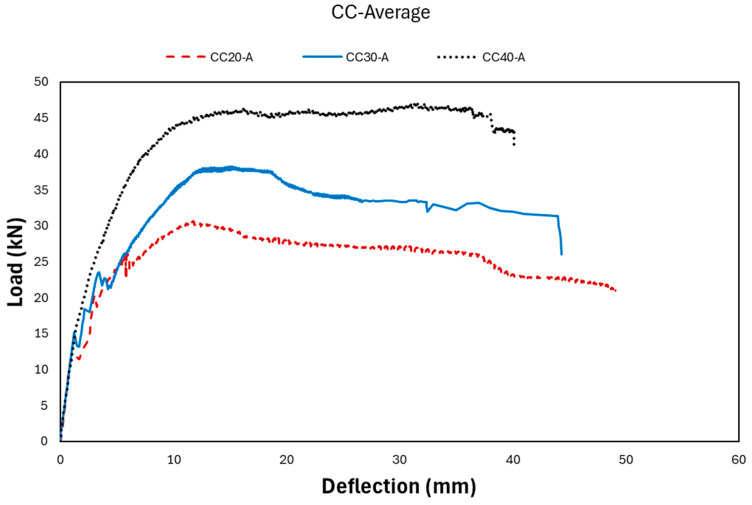
Load-deflection results of GFRP RC beams [3].

The effectiveness of GFRP bars in the tension zone of reinforced concrete (RC) beams was investigated at three different concrete strength levels: low (20 MPa), medium (30 MPa), and high (40 MPa). The average load–deflection values obtained from the experiments are presented in Figure 3 [3].

Based on the results of the three-point bending tests, the effect of concrete compressive strength on the load-carrying capacity and deflection behavior of GFRP-reinforced concrete beams is clearly observed. Specifically, with increasing concrete compressive strength, the load-carrying capacity of the RC beams increased, while the corresponding deflection values decreased. This indicates a reduction in ductility and an increase in stiffness with higher concrete strength. The experimentally obtained load–deflection data consist of thousands of data points for each specimen. These data were divided into training and testing datasets using an 80–20% split. In this study, the failure condition was specifically analyzed because the maximum deflection capacity and load levels that GFRP-reinforced beams can reach, particularly under the influence of extreme forces, were investigated. Based on the results obtained, the applicability and performance of GFRP reinforcement were evaluated.

Another important aspect of the beam failure stage is related to structural engineering requirements. In structural design, ductile structural behavior is generally preferred over sudden brittle failure under applied loads. Therefore, it is desirable for beam elements to exhibit ductile behavior rather than brittle behavior when approaching the ultimate state.

In addition, the proposed method was used to investigate how much deflection the beams may experience under different load levels and which parameters become dominant in controlling this behavior. These parameters may vary depending on the material strength class. For example, in beams with lower concrete compressive strength, concrete compressive strength significantly influences structural behavior by increasing the load-carrying capacity while reducing the deflection capacity and ductility. The deflection behavior of FRP-reinforced beams is often governed by serviceability considerations due to their relatively lower stiffness compared with steel reinforcement. Therefore, in this study, the deflection capacity and load–deflection behavior of GFRP-reinforced beams were investigated using experimental data, with the aim of contributing to the literature and demonstrating the applicability of innovative technologies in this field. Higher concrete compressive strength leads to a higher elastic modulus of concrete, which increases the flexural rigidity of the beam and results in a steeper initial slope in the load–deflection response. This trend can be observed in the experimental curves, where the C40 beams show higher stiffness compared to C30 and C20 beams. In addition, higher-strength concrete generally delays the formation of flexural cracks due to its improved tensile capacity, which allows the beam to maintain its uncracked stiffness for a longer loading stage. The crack images [3] support this observation, as beams with lower compressive strength exhibit earlier crack initiation and wider crack propagation, while higher-strength beams show delayed and more localized cracking near mid-span.

After cracking, stiffness decreases due to the reduction in the effective moment of inertia. However, beams with higher concrete compressive strength maintain relatively better stiffness and can sustain higher loads before reaching their ultimate deformation. This behavior is particularly important for GFRP-reinforced beams, where the relatively low modulus of elasticity of GFRP makes the deflection response more sensitive to the stiffness contribution of the concrete section.

### 3.2. SHAP Analysis

In the present study, a comprehensive set of material, geometric, and structural parameters of GFRP-reinforced concrete beams were considered as input variables for the machine learning-based SHAP analysis to evaluate and interpret the deflection behavior. The material properties included concrete compressive strength (fck), concrete tensile strength (fct), and concrete modulus of elasticity (Ec), as well as the reinforcement ratio (ρf) and modulus of elasticity of the GFRP reinforcement (Ef). In addition, the geometric properties of the beams, namely beam width (b), beam height (h), span length (L), and effective depth (d), were incorporated into the analysis. Furthermore, structural response and performance-related parameters, including the initial cracking load (Fcr), experimental failure moment (Mexp), effective moment of inertia (Ieff), applied load (P), slenderness ratio (L/d), creep coefficient under long-term loading conditions (θ), cracking ratio (M/Mcr), stirrup spacing, longitudinal reinforcement diameter, and shear span-to-depth ratio (a/d), were also considered. These input variables were utilized within the SHAP-based machine learning framework to quantify and interpret their relative contributions and influence on the deflection response of GFRP-reinforced concrete beams. Detailed properties of the beams corresponding to the average load–deflection responses shown in Figure 3 are presented in Table 3.

**Figure 4:**
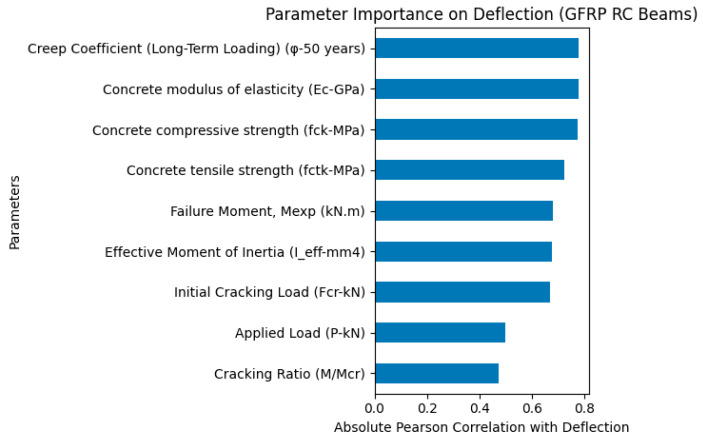
Overall parameter importance for deflection of GFRP-reinforced concrete beams.

The parameter importance analysis presented in Figure 4 reveals that the creep coefficient under long-term loading conditions (φ) is the most influential parameter governing the deflection behavior of GFRP-reinforced concrete beams with GFRP bars in the tension zone. This finding highlights the dominant role of time-dependent deformation effects in controlling beam deflection, indicating that long-term creep significantly contributes to stiffness degradation and increased deformation capacity. In addition, the concrete modulus of elasticity (Ec) and concrete compressive strength (fck) were identified as highly influential parameters, confirming that the elastic stiffness and mechanical properties of concrete play a critical role in resisting deformation.

Furthermore, the concrete tensile strength (fctk), effective moment of inertia (Ieff), and failure moment (Mexp) also exhibited substantial influence, emphasizing the importance of cracking behavior and post-cracking flexural stiffness in the deflection response. The initial cracking load (Fcr), applied load (P), and cracking ratio (M/Mcr) showed moderate influence, indicating that both load level and cracking state contribute to deformation characteristics. Overall, the results presented in Figure 4 demonstrate that the deflection behavior of tension zone GFRP-reinforced RC beams is primarily governed by long-term creep effects and concrete stiffness-related parameters, confirming that both time-dependent behavior and flexural rigidity are key factors controlling beam deflection.

**Figure 5:**
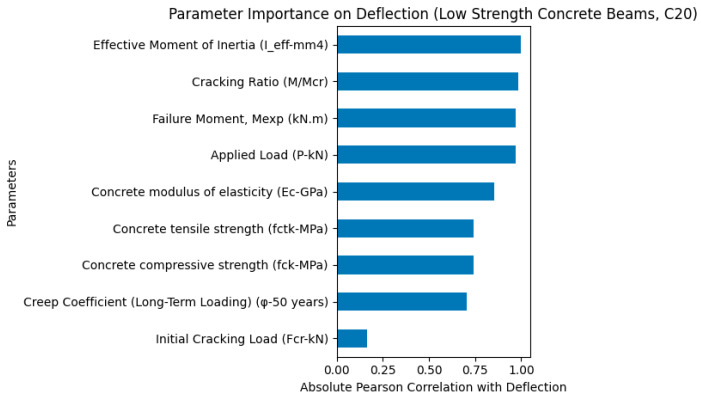
Parameter importance analysis for deflection of low-strength (C20) concrete GFRP-RC beams.

It should be noted that the geometric parameters, including beam width, beam height, span length, and effective depth, showed negligible influence on the deflection response. This is primarily because these parameters were kept constant for all specimens considered in this study. As a result, no variability existed in these features within the dataset, and therefore their contribution to the deflection prediction and importance ranking was insignificant. While Figure 4 presents the parameter importance results obtained by analyzing the complete dataset of all beam specimens, Figure 5 illustrates the parameter importance analysis specifically for low-strength concrete beams (C20 series). As shown in Figure 5, the parameter importance analysis for low-strength concrete beams indicates that the effective moment of inertia (Ieff) is the most influential parameter governing deflection behavior. This highlights the dominant role of flexural stiffness degradation after cracking in controlling the deformation response of GFRP-reinforced concrete beams with low concrete strength. Furthermore, cracking-related parameters such as cracking ratio (M/Mcr) and failure moment (Mexp), along with the applied load (P), also exhibited significant influence. In contrast, concrete mechanical properties and creep coefficient showed relatively lower influence compared to stiffness-related parameters, indicating that the deflection behavior of low-strength concrete beams is primarily governed by cracking-induced stiffness reduction rather than long-term creep effects.

**Figure 6:**
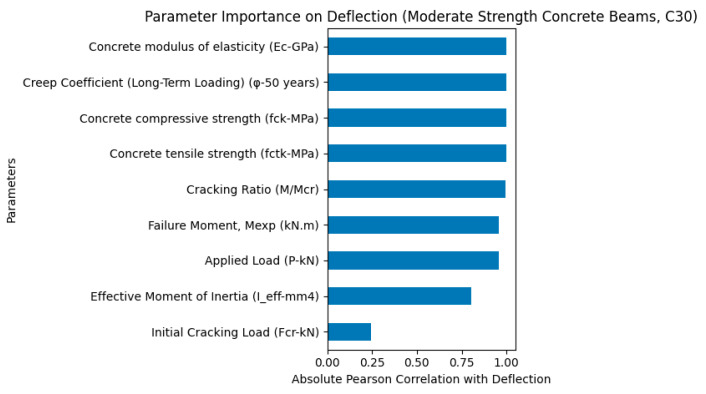
Parameter importance analysis for deflection of moderate-strength (C30) concrete GFRP-RC beams.

As shown in Figure 6, the parameter importance analysis for moderate-strength concrete beams (C30 series) indicates that the concrete modulus of elasticity (Ec), creep coefficient (φ), concrete compressive strength (fck), concrete tensile strength (fctk), and cracking ratio (M/Mcr) are the most influential parameters governing the deflection response. Among these, the concrete modulus of elasticity and creep coefficient exhibited the highest influence, highlighting the dominant role of both instantaneous elastic stiffness and long-term time-dependent deformation in controlling deflection behavior.

Furthermore, flexural performance-related parameters such as failure moment (Mexp), applied load (P), and effective moment of inertia (Ieff) also showed significant contributions, indicating that both structural stiffness and load level play important roles in determining beam deformation. In contrast, the initial cracking load (Fcr) showed relatively lower influence compared to other parameters. Overall, these results demonstrate that the deflection behavior of GFRP-reinforced concrete beams with moderate concrete strength is governed by a combined effect of concrete stiffness, creep behavior, and flexural rigidity.

**Figure 7:**
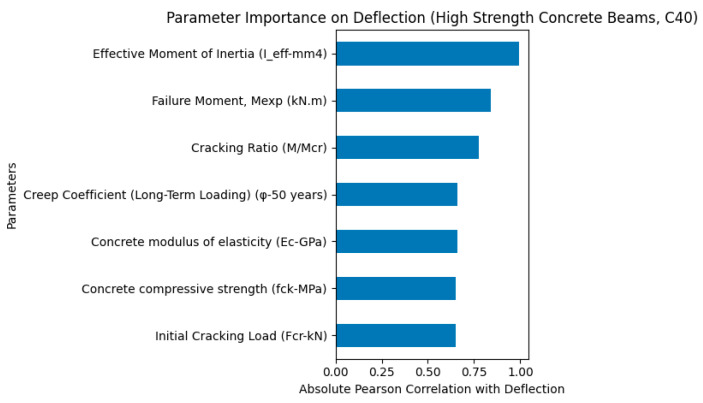
Parameter importance analysis for deflection of moderate-strength (C40) concrete GFRP-RC beams.

As shown in Figure 7, the parameter importance analysis for high-strength concrete beams (C40 series) indicates that the effective moment of inertia (Ieff) is the most influential parameter governing the deflection behavior. This highlights the dominant role of flexural stiffness in controlling the deformation response of GFRP-reinforced concrete beams with high concrete strength. In addition, the failure moment (Mexp) and cracking ratio (M/Mcr) also exhibited significant influence, indicating that flexural capacity and cracking behavior remain critical factors affecting deflection.

Furthermore, the creep coefficient (φ), concrete modulus of elasticity (Ec), and concrete compressive strength (fck) showed moderate influence, suggesting that both long-term deformation effects and concrete mechanical stiffness contribute to the overall deflection response. The initial cracking load (Fcr) also showed a noticeable influence, reflecting the importance of crack initiation behavior in high-strength concrete beams. Overall, the results demonstrate that the deflection behavior of high-strength concrete GFRP-reinforced beams is primarily governed by flexural stiffness and cracking-related parameters, with reduced relative influence of creep compared to lower-strength concrete beams.

All parameter importance plots were produced using the Python programming environment (Python 3.11). Data preprocessing and analysis were performed using the Pandas library (v2.2), and graphical visualizations were generated using the Matplotlib library (v3.8).

**Figure 8:**
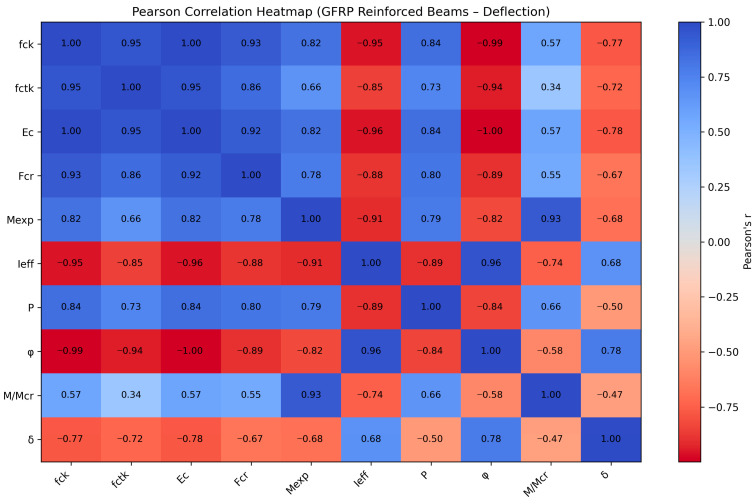
Pearson correlation heatmap of parameters affecting deflection (δ) in GFRP-reinforced beams.

As shown in Figure 8, the Pearson correlation heatmap reveals the strength and direction of linear relationships between the governing parameters and the measured deflection (δ) of GFRP-reinforced concrete beams. A strong positive correlation is observed between the creep coefficient (φ) and deflection (r = 0.78), indicating that long-term creep effects significantly increase beam deflection. This finding is consistent with the viscoelastic behavior of concrete, where higher creep leads to greater time-dependent deformation.

Conversely, strong negative correlations are observed between deflection and the concrete mechanical properties, particularly the modulus of elasticity (Ec) (r = −0.78) and compressive strength (fck) (r = −0.77). These results indicate that higher stiffness and strength of concrete effectively reduce deflection by increasing the overall flexural rigidity of the beam. Similarly, the cracking load (Fcr) and failure moment (Mexp) also exhibit negative correlations with deflection (r = −0.67 and r = −0.68, respectively), suggesting that structurally stronger beams with higher cracking and ultimate capacities experience lower deflections.

In addition, the effective moment of inertia (Ieff) shows a positive correlation with deflection (r = 0.68), which reflects the interaction between stiffness degradation and deformation behavior in cracked sections. The cracking ratio (M/Mcr) demonstrates a moderate negative correlation (r = −0.47), indicating its secondary but still meaningful influence on deflection performance.

Collectively, Figure 8 confirms that the creep coefficient, elastic modulus, compressive strength, and flexural capacity parameters are the most influential variables governing deflection in GFRP-reinforced beams. These results are consistent with established structural mechanical principles and highlight the dominant role of material stiffness and long-term deformation effects in controlling serviceability performance.

The failure moment influences the deflection of reinforced concrete (RC) beams because it is directly related to the beam’s flexural capacity, stiffness degradation, and cracking behavior under increasing loads.

As the applied bending moment approaches the failure moment, the beam experiences progressive cracking and stiffness reduction. In RC beams, especially those reinforced with GFRP bars, the stiffness decreases significantly after cracking because GFRP reinforcement has a lower modulus of elasticity compared to steel reinforcement. This leads to larger curvature and consequently greater deflection under the same load level.

Mechanically, deflection in beams is governed by the moment–curvature relationship and the effective moment of inertia (I_eff). As the bending moment increases toward the failure moment, the effective stiffness (EI) of the beam decreases due to crack propagation and redistribution of stresses between concrete and reinforcement. This reduction in stiffness results in larger curvature along the span, which ultimately increases the beam deflection.

Therefore, the failure moment becomes an important parameter because it reflects the ultimate flexural resistance and the extent of stiffness degradation before failure, both of which significantly influence the deflection behavior of RC beams. In GFRP-reinforced beams, this effect is even more pronounced due to their higher deformability and reduced post-cracking stiffness compared to conventional steel-reinforced beams.

Concrete compressive strength significantly affects the flexural stiffness and cracking response of reinforced concrete beams through its relationship with the elastic modulus of concrete (E_c) and the cracking moment (M_cr). As the compressive strength increases, the modulus of elasticity of concrete also increases, which leads to greater initial flexural stiffness (EI) of the beam. Consequently, beams made with higher-strength concrete exhibit lower deflection under the same load levels during the pre-cracking stage.

Moreover, higher concrete compressive strength increases the tensile strength of concrete, which delays the formation of the first flexural cracks and increases the cracking moment (M_cr). This delay in crack initiation results in a longer uncracked stiffness stage, thereby improving the overall stiffness performance of the beam. After cracking occurs, the stiffness gradually decreases due to the reduction in the effective moment of inertia; however, beams with higher compressive strength generally maintain greater post-cracking stiffness compared to beams with lower concrete strength.

The experimental load–deflection curves presented in the study support this behavior. As illustrated in the figure, the beam with C40 concrete (CC40-A) demonstrates the highest load capacity and stiffness, while the C30 and C20 beams show progressively lower stiffness and earlier stiffness degradation. The CC40 beam sustains higher loads with relatively smaller deflection in the early loading stages, indicating enhanced stiffness and delayed cracking behavior compared to the lower-strength concrete beams.

These observations are also consistent with previous studies reported in the literature, which indicate that increasing concrete compressive strength improves flexural rigidity, delays crack initiation, and enhances the serviceability performance of reinforced concrete beams, particularly in beams reinforced with GFRP bars where serviceability and stiffness play a critical role.

**Figure 9:**
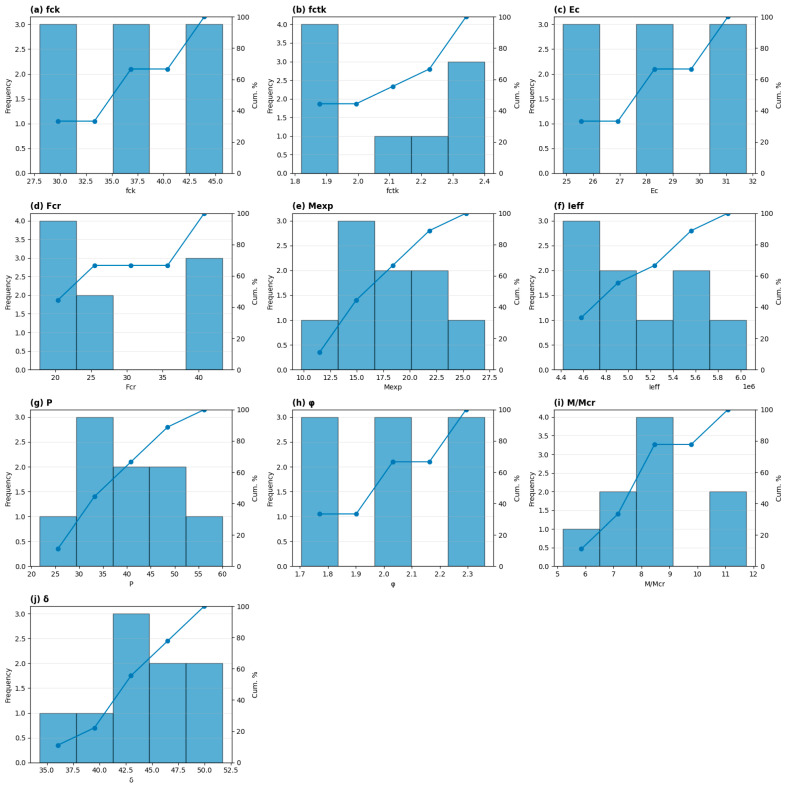
Frequency and cumulative percent distributions of parameters affecting deflection in GFRP-reinforced beams.

Figure 9 illustrates the frequency and cumulative percent distributions of the main material and structural parameters affecting the deflection behavior of GFRP-reinforced concrete beams. The frequency bars represent the number of specimens falling within each parameter interval, while the cumulative percent curves indicate the progressive percentage of specimens up to a given parameter value, providing insight into the statistical distribution and variability of each variable.

As shown in Figure 9a–c, the concrete mechanical properties, including compressive strength (fck), tensile strength (fctk), and elastic modulus (Ec), exhibit relatively narrow distributions, indicating consistent material properties across the tested specimens. The cumulative percent curves for these parameters increase steadily, reaching approximately 100% within limited value ranges, which confirms low dispersion and good uniformity of concrete properties.

Figure 9d,e shows the distributions of cracking load (Fcr) and failure moment (Mexp), which exhibit wider spreads compared to basic material properties. The gradual slope of the cumulative percent curves indicates greater variability in structural capacity, reflecting differences in cracking resistance and ultimate flexural performance among the specimens. This variability directly influences the structural stiffness and deflection response.

In Figure 9f–h, the effective moment of inertia (Ieff), applied load (P), and creep coefficient (φ) demonstrate moderate to wide distributions. The cumulative percent curves for these parameters show non-uniform slopes, indicating heterogeneous structural stiffness and loading conditions. In particular, the creep coefficient exhibits a relatively broad range, confirming the significant influence of long-term deformation effects on beam behavior.

Figure 9i,j presents the distributions of cracking ratio (M/Mcr) and deflection (δ). The deflection values are distributed over a moderate range, and the cumulative percent curve shows a gradual increase, indicating that deflection varies consistently with changes in stiffness, loading, and creep parameters. The numerical values shown in the cumulative percent curves represent the proportion of specimens that fall below or equal to a given parameter value. For example, a cumulative percent of 60% at a specific deflection level indicates that 60% of the tested beams exhibit deflection values equal to or less than that level.

Figure 9 provides a comprehensive statistical representation of the parameter distributions and demonstrates that material stiffness, structural capacity, and loading conditions play a dominant role in governing the variability of deflection in GFRP-reinforced concrete beams. In addition, the distribution of the creep coefficient (φ) highlights the influence of time-dependent deformation effects, confirming that long-term creep behavior contributes significantly to the observed deflection response.

**Figure 10:**
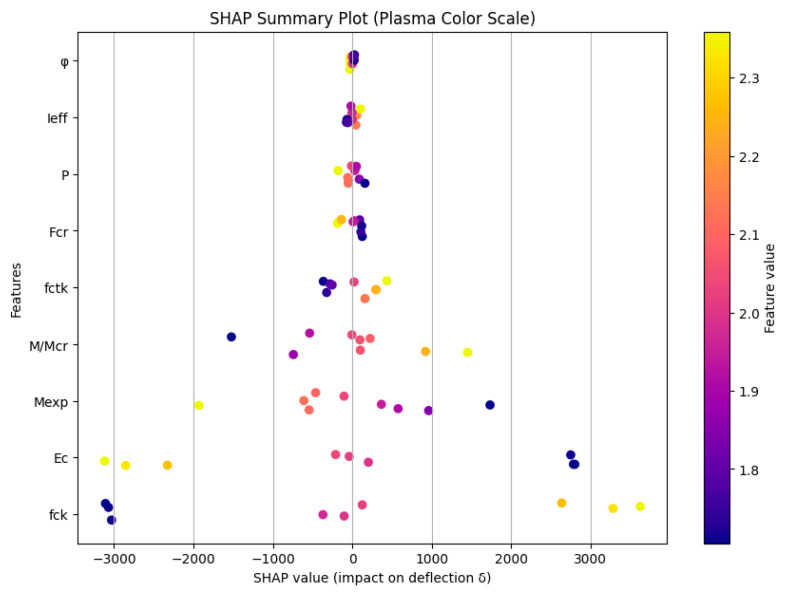
SHAP summary plot of parameters affecting deflection (δ) in GFRP-reinforced beams.

Figure 10 presents the SHAP summary plot illustrating the relative importance and directional influence of the input parameters on the deflection (δ) of GFRP-reinforced concrete beams. In this plot, each point represents an individual specimen, while the horizontal position indicates the SHAP value, which quantifies the contribution of a given parameter to increasing or decreasing the predicted deflection. Positive SHAP values correspond to parameters that increase deflection, whereas negative SHAP values indicate parameters that reduce deflection. The color scale represents the magnitude of the parameter value, with warmer colors indicating higher values and cooler colors representing lower values.

As shown in Figure 10, the creep coefficient (φ) exhibits the highest overall influence on deflection, as indicated by its wide SHAP value distribution and dominant position in the ranking. Higher values of φ are generally associated with positive SHAP values, confirming that increased creep significantly contributes to higher deflection due to time-dependent deformation effects. Similarly, the effective moment of inertia (Ieff) and applied load (P) demonstrate substantial influence on deflection. Higher applied load values tend to increase deflection, whereas higher effective stiffness conditions are associated with reduced deflection depending on the structural response.

The concrete mechanical properties, including compressive strength (fck), tensile strength (fctk), and elastic modulus (Ec), also show significant contributions. In particular, higher values of Ec and fck are predominantly associated with negative SHAP values, indicating that increased material stiffness reduces deflection by enhancing flexural rigidity. The failure moment (Mexp) and cracking load (Fcr) exhibit moderate influence, reflecting their role in governing structural capacity and stiffness degradation after cracking.

Figure 10 confirms that both material stiffness parameters and time-dependent creep effects play a critical role in controlling the deflection behavior of GFRP-reinforced beams. The SHAP analysis provides clear quantitative evidence of the relative importance and directional effects of each parameter, highlighting creep coefficient, stiffness-related properties, and applied load as the dominant factors influencing deflection performance.

**Figure 11:**
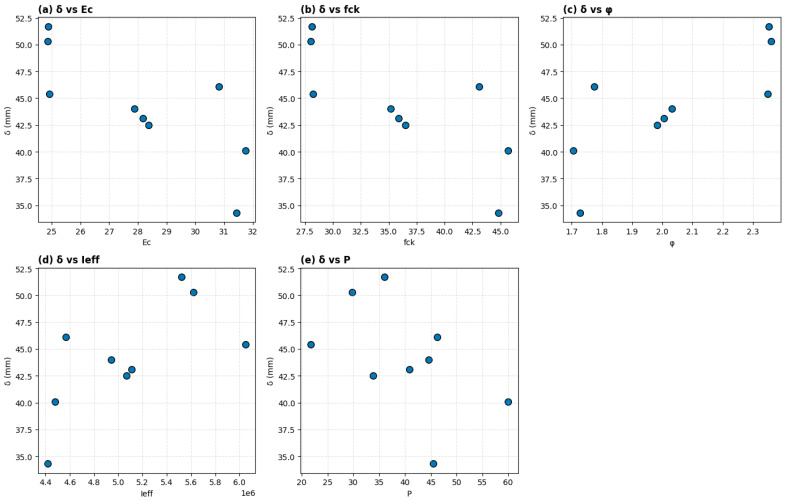
Scatter plots of deflection (δ) versus key parameters in GFRP-reinforced concrete beams.

In Figure 11, the numerical values on both the horizontal and vertical axes represent the measured magnitudes of the structural parameters and the corresponding deflection values for each tested GFRP-reinforced concrete beam specimen.

Specifically, the horizontal axis in each subplot represents the actual measured value of the corresponding parameter, while the vertical axis represents the measured deflection (δ) in millimeters. Each individual point corresponds to one beam specimen, and its position indicates the combination of parameter value and resulting deflection for that specimen.

For example, in Figure 11a, the horizontal axis values represent the concrete elastic modulus (Ec) in GPa, while the vertical axis values represent the corresponding deflection in mm. A point located at Ec = 25 GPa and δ = 51 mm indicates that a beam with an elastic modulus of 25 GPa experienced a deflection of 51 mm. Similarly, in Figure 11b, a point at fck = 45 MPa and δ = 40 mm indicates that a specimen with a compressive strength of 45 MPa exhibited a deflection of 40 mm.

In Figure 11c, the horizontal axis values represent the creep coefficient (φ), which is dimensionless, while the vertical axis represents deflection in mm. Higher φ values correspond to greater long-term deformation effects. In Figure 11d, the horizontal axis represents the effective moment of inertia (Ieff) in mm^4^, which quantifies the flexural stiffness of the beam section, and higher Ieff values generally correspond to improved stiffness characteristics. In Figure 11e, the horizontal axis represents the applied load (P) in kN, and the vertical axis shows the resulting deflection in mm.

The numerical values therefore represent real experimental measurements and allow direct visualization of how changes in each parameter influence the magnitude of deflection. The relative position and spread of the points indicate the strength, direction, and sensitivity of the relationship between each parameter and deflection.

**Figure 12:**
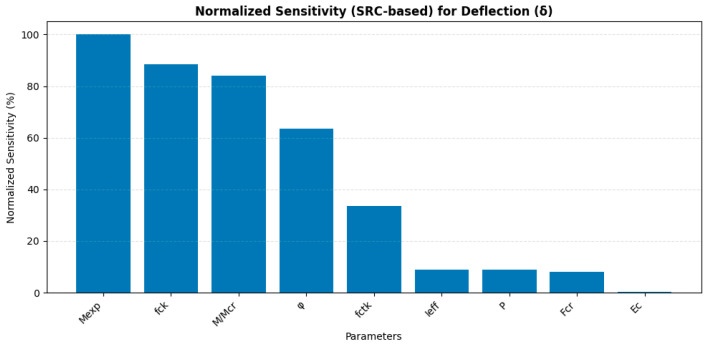
Normalized sensitivity analysis of parameters affecting deflection (δ) in GFRP-reinforced concrete beams.

Figure 12 presents the normalized sensitivity analysis of the input parameters with respect to the deflection (δ) of GFRP-reinforced concrete beams based on standardized regression coefficients (SRC). The horizontal axis shows the structural and material parameters using their symbolic notation, while the vertical axis represents the normalized sensitivity expressed as a percentage (%). These values quantify the relative contribution of each parameter to the variation in deflection, where 100% corresponds to the most influential parameter and lower percentages indicate proportionally smaller influence. As shown in Figure 12, the failure moment (Mexp) exhibits the highest normalized sensitivity, reaching 100%, indicating that it is the most critical parameter influencing deflection within the analyzed dataset. This result suggests that the flexural capacity of the beam plays a dominant role in governing deformation behavior. The concrete compressive strength (fck) and cracking ratio (M/Mcr) also demonstrate high sensitivity levels, approximately 89% and 84%, respectively, confirming their significant influence on structural stiffness and cracking behavior, which directly affect deflection. The creep coefficient (φ) shows moderate sensitivity, approximately 64%, indicating that time-dependent deformation contributes substantially to deflection variation. The tensile strength of concrete (fctk) exhibits lower sensitivity, around 34%, suggesting a secondary influence compared to compressive strength and creep effects. In contrast, the effective moment of inertia (Ieff), applied load (P), and cracking load (Fcr) show relatively low sensitivity values, each below approximately 10%, indicating a limited influence on deflection variation within the specific range of the dataset.

Finally, the elastic modulus of concrete (Ec) exhibits the lowest normalized sensitivity, approaching 0%, suggesting minimal contribution to deflection variability under the given conditions. Overall, Figure 12 provides a quantitative ranking of the parameters affecting deflection and identifies the failure moment, compressive strength, cracking ratio, and creep coefficient as the most influential variables governing the deflection behavior of GFRP-reinforced concrete beams.

## 4. Discussion

The comparative parameter importance analyses for low-strength (C20), moderate-strength (C30), and high-strength (C40) concrete beams reveal distinct differences in load-carrying capacity, ductility, and deflection behavior of GFRP-reinforced concrete beams.

Firstly, the load-carrying capacity increased significantly with increasing concrete compressive strength. The C40 beams exhibited the highest flexural capacity, followed by the C30 and C20 beams. This is primarily attributed to the improved compressive resistance and higher stiffness of high-strength concrete, which enhances the overall flexural rigidity and load resistance of the beam system.

In terms of deflection behavior, the C20 beams exhibited the highest deflection values, indicating lower stiffness and greater deformability. This behavior reflects a more ductile structural response, as the beams undergo larger deformations before reaching their ultimate capacity. The parameter importance analysis confirmed that, in C20 beams, deflection was mainly governed by stiffness degradation after cracking, particularly influenced by the effective moment of inertia and cracking ratio. This indicates that low-strength concrete beams are more sensitive to stiffness reduction caused by crack propagation.

In contrast, the C40 beams exhibited the lowest deflection values, indicating significantly higher stiffness and reduced deformation capacity. The importance analysis showed that deflection in C40 beams was strongly governed by the effective moment of inertia and flexural capacity, reflecting a stiffness-controlled response. However, despite their higher load-carrying capacity, the reduced deformation capacity indicates a more brittle structural behavior compared to lower-strength concrete beams. This is consistent with the known behavior of high-strength concrete, which exhibits limited strain capacity and reduced ductility.

The C30 beams demonstrated intermediate behavior between C20 and C40 beams in terms of both load-carrying capacity and deflection response. The parameter importance analysis indicated that both stiffness-related parameters and creep effects contributed significantly to deflection behavior, suggesting a balanced structural response combining stiffness and deformation capacity.

Overall, the results demonstrate that increasing concrete strength leads to increased load-carrying capacity and stiffness, but reduced deflection capacity and ductility. The C20 beams exhibited more ductile behavior with higher deflection capacity, while the C40 beams exhibited higher stiffness and load capacity but more brittle structural behavior. The C30 beams showed a transition behavior between ductile and brittle responses. These findings confirm that concrete compressive strength plays a critical role in controlling the stiffness, ductility, and deflection performance of GFRP-reinforced concrete beams.

The variation in parameter importance observed across Pearson correlation, SHAP analysis, scatter plots, and sensitivity analysis is primarily attributed to the different mathematical foundations and evaluation criteria of each method. Pearson correlation quantifies the strength of linear association between individual input parameters and deflection without considering the combined influence of other variables. In contrast, sensitivity analysis using standardized regression coefficients evaluates the relative influence of each parameter within a multivariate predictive framework, where the effect of each variable is assessed while accounting for the presence of other parameters. Similarly, SHAP analysis provides a model-based interpretation by quantifying the contribution of each parameter to the prediction outcome, considering both the magnitude and interaction effects within the model.

Within the scope of this study, parameters such as reinforcement ratio and span were not considered as variable parameters. Since these values were kept constant for all beams, it would not be appropriate to compare their influence on deflection; therefore, no interpretation regarding these parameters was included. However, detailed analyses regarding material properties—particularly concrete compressive strength—are provided. Moreover, as shown in Figure 3, even the increase in concrete compressive strength alone leads to an increase in both the ultimate load-carrying capacity and the initial stiffness of the beams, while the corresponding deflection values decrease.

These methodological differences explain why certain parameters, such as the creep coefficient (φ), elastic modulus (Ec), compressive strength (fck), and failure moment (Mexp), may exhibit varying importance rankings across different analyses. Additionally, the presence of interdependence among structural parameters, such as the inherent relationship between compressive strength, elastic modulus, and flexural capacity, can redistribute the relative contribution of each parameter depending on the analytical approach used. Furthermore, the limited sample size and relatively narrow variation ranges of some parameters may also influence the sensitivity and relative importance rankings.

Despite these differences, a consistent trend can be identified when all analytical methods are considered collectively. The results consistently indicate that stiffness-related parameters and flexural capacity parameters play a dominant role in governing deflection behavior in GFRP-reinforced concrete beams.

Based on the combined evaluation of all analytical methods, the most influential parameters affecting deflection can be ranked as follows:

➢ Creep coefficient (φ)—Most critical parameter due to its strong influence on time-dependent deflection;
➢ Elastic modulus of concrete (Ec)—Governs flexural stiffness and deformation resistance;
➢ Compressive strength of concrete (fck)—Directly affects structural stiffness and cracking behavior;
➢ Failure moment (Mexp)—Represents the flexural capacity and structural resistance;
➢ Effective moment of inertia (Ieff)—Controls sectional stiffness after cracking;
➢ Applied load (P)—Directly influences the magnitude of deflection;
➢ Cracking-related parameters (Fcr and M/Mcr)—Influence stiffness degradation and structural response.

**Figure 13:**
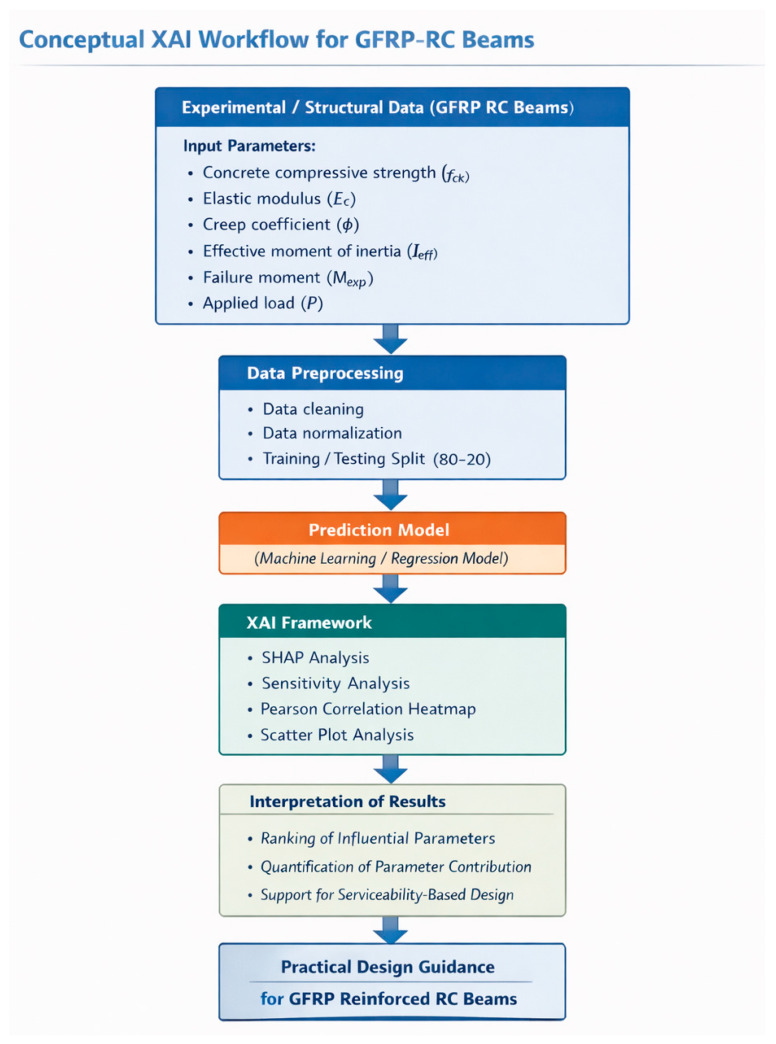
Conceptual XAI workflow for deflection prediction in GFRP RC beams.

These results confirm that the deflection behavior of GFRP-reinforced concrete beams is primarily governed by stiffness-related parameters, flexural capacity, and time-dependent deformation effects, highlighting the critical role of both material properties and structural characteristics in controlling serviceability performance. Figure 13 presents conceptual XAI workflow for predicting and interpreting the deflection behavior of GFRP RC beams, integrating experimental data, machine learning prediction, and explainable AI analyses to identify the influence of governing parameters.

## 5. Conclusions

The results indicate that GFRP reinforcement is less effective in controlling deflection in low-strength concrete beams due to insufficient stiffness, while its effectiveness improves in medium-strength concrete and becomes significantly more efficient in high-strength concrete beams, where increased concrete stiffness enhances composite action and reduces deflection.

Concrete compressive strength significantly increases load-carrying capacity while reducing deflection capacity and ductility.

Effective moment of inertia (Ieff) is the most critical parameter controlling deflection behavior, particularly in low- and high-strength concrete beams.

Concrete modulus of elasticity (Ec) strongly governs beam stiffness and plays a dominant role in reducing deflection.

Creep coefficient (φ) has a major influence on deflection, especially when all beam groups are considered together, confirming the importance of long-term deformation effects.

Low-strength concrete beams (C20) exhibit higher deflection and more ductile behavior due to lower stiffness and earlier stiffness degradation after cracking.

High-strength concrete beams (C40) exhibit lower deflection and higher load capacity but more brittle structural behavior due to increased stiffness and reduced deformation capacity.

Moderate-strength concrete beams (C30) show intermediate behavior, representing a transition between ductile and brittle response.

Cracking-related parameters, particularly cracking ratio (M/Mcr) and failure moment (Mexp), significantly influence deflection by controlling post-cracking stiffness.

Applied load (P) has a considerable influence on deflection, especially in lower-strength concrete beams.

Geometric and reinforcement parameters showed negligible influence because they were kept constant across all specimens.

It is recommended that future research focus on the variability of geometric parameters, especially across different concrete strength classes, to provide a more comprehensive understanding of their influence on the deflection response and structural behavior of GFRP-reinforced concrete beams.

A similar study could be conducted for UHPC beams reinforced with FRP longitudinal reinforcement to investigate their deflection behavior and the influence of governing parameters.

## Figures and Tables

**Table 1:** Mechanical characteristics of GFRP bars [4].

GFRP Specimen Diameter (mm)	Cross Sectional Area (mm^2^)	Ultimate Tensile Strength (MPa)	Ultimate Bending Load (kN)	Ultimate Bending Strength (MPa)	Max. Deflection Δ (mm)	Weight (gr/cm)	Support Distance (mm)
10	78.54	810	3.2	40.11	4.67	1.59	100

**Table 2:** Mechanical characteristics of concrete groups.

Concrete Group	fc (Cylinder-MPa)	fctk (MPa)
C20	28.1	1.85
C30	35.9	2.10
C40	44.8	2.30

**Table 3:** Data used in the SHAP analysis.

		Specimens								
		C20-1	C20-2	C20-3	C30-1	C30-2	C30-3	C40-1	C40-2	C40-3
Input	Concrete compressive strength (fck-MPa)	28.1	28	28.2	35.9	36.5	35.2	44.8	45.7	43.1
	Concrete tensile strength (fctk-MPa)	1.85	1.82	1.88	2.1	2.2	1.9	2.3	2.4	2.3
	Concrete modulus of elasticity (Ec-GPa)	24.89	24.87	24.93	28.17	28.37	27.89	31.44	31.75	30.83
	Reinforcement ratio (ρf)	0.00717	0.00717	0.00717	0.00717	0.00717	0.00717	0.00717	0.00717	0.00717
	Modulus of Elasticity of Reinforcement (Ef-GPa)	50	50	50	50	50	50	50	50	50
	Beam width (b-mm)	150	150	150	150	150	150	150	150	150
	Beam height (h-mm)	200	200	200	200	200	200	200	200	200
	Span length (L-mm)	900	900	900	900	900	900	900	900	900
	Effective depth (d-mm)	146	146	146	146	146	146	146	146	146
	Initial Cracking Load (Fcr-kN)	20.47	17.76	19.23	18.35	25.98	27.23	41.93	43.37	39.22
	Failure Moment, Mexp (kN.m)	16.2	13.4	9.77	18.41	15.21	20.09	27	20.48	20.79
	Effective Moment of Inertia (I_eff-mm^4^)	5.52 × 10^6^	5.62 × 10^6^	6.05 × 10^6^	5.11 × 10^6^	5.07 × 10^6^	4.94 × 10^6^	4.42 × 10^6^	4.48 × 10^6^	4.57 × 10^6^
	Applied Load (P-kN)	36.01	29.71	21.7	40.91	33.8	44.64	45.5	60.01	46.2
	Slenderness Ratio (L/d)	6.164	6.164	6.164	6.164	6.164	6.164	6.164	6.164	6.164
	Creep Coefficient (Long-Term Loading) (φ-50 years)	2.353	2.359	2.348	2.005	1.983	2.031	1.728	1.705	1.774
	Cracking Ratio (M/Mcr)	8.757	7.363	5.197	8.767	6.914	10.574	11.739	8.533	9.039
	Stirrup spacing (mm)	300	300	300	300	300	300	300	300	300
	Longitudinal Rebar diameter (mm)	10	10	10	10	10	10	10	10	10
	Shear span-to-depth ratio (a/d-mm/mm)	2.055	2.055	2.055	2.055	2.055	2.055	2.055	2.055	2.055
Output	Deflection (δ-mm)	51.7	50.3	45.4	43.1	42.5	44	34.3	40.1	46.1

## Data Availability

The raw data supporting the conclusions of this article will be made available by the author on request.
